# 4D-Spatiotemporal SHG Imaging for the Analysis of Drug-Induced Changes in the Dura Mater

**DOI:** 10.1021/acs.analchem.4c04887

**Published:** 2025-02-14

**Authors:** Constanze Schultz, Marko Rodewald, Andreas Weidisch, Tobias Meyer-Zedler, Thomas Caffard, Michael Schmitt, Georg Matziolis, Timo Zippelius, Jürgen Popp

**Affiliations:** † Leibniz Institute of Photonic Technology (Leibniz-IPHT), Member of Leibniz Health Technologies, Member of the Leibniz Center for Photonics in Infection Research (LPI), Albert-Einstein-Straße 9, 07745 Jena, Germany; ‡ Orthopedic Department, Jena University Hospital, Campus Eisenberg, Klosterlausnitzer Straße 81, 07607 Eisenberg, Germany; § Institute of Physical Chemistry (IPC) and Abbe Center of Photonics (ACP), Member of the Leibniz Center for Photonics in Infection Research (LPI), Friedrich Schiller University Jena, Helmholtzweg 4, 07743 Jena, Germany; ∥ Department of Orthopedic Surgery, University of Ulm, Oberer Eselsberg 45, 89081 Ulm, Germany; ⊥ Cluster of Excellence Balance of the Microverse, Friedrich Schiller University Jena, Fürstengraben 1, 07743 Jena, Germany

## Abstract

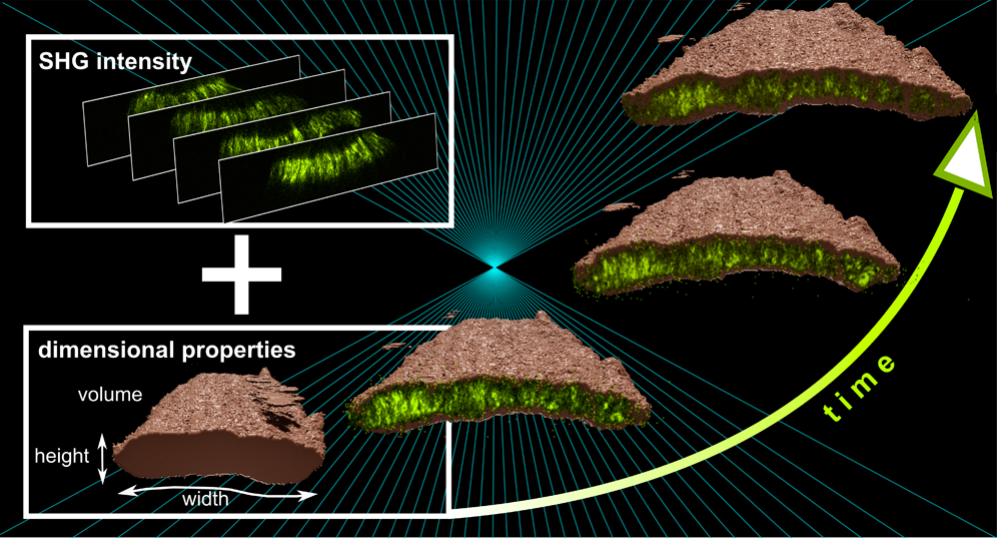

The spatiotemporal assessment of tissue dynamics after the introduction of disruptive factors is crucial for evaluating their impact and for developing effective countermeasures. Here, we report a 4D-spatiotemporal imaging approach using second harmonic generation (SHG) imaging microscopy, enabling an advanced time-resolved analysis of three-dimensional tissue features. This is of particular interest as topical administration of drugs during spinal surgeries is a standard practice for preventing and treating postoperative complications like infections. Local drug concentrations on tissue are high in these scenarios, and given the dura’s role as a protective barrier for the brain and spinal cord, potential drug-induced damage should be evaluated critically. By employing 4D-SHG imaging, we gained detailed insights into changes in dimensional properties of thin section samples, namely, width, height, and volume, as well as into alterations within the hierarchic structure of collagen. The latter thereby allowed us to postulate a mode of action, which we attributed for the herein investigated samples to the pH of the formulation.

The evaluation of tissue morphology and composition is a fundamental and recurring task in biomedical research. Great progress has been achieved in that regard within the last decades concerning the use of nonlinear imaging techniques, e.g., as a histopathologic tool.^1−3^ Although appearing static at first glance, microscopic images are merely snapshots of inherently dynamic processes, which unfold over time in somewhat complex spatial environments. For slow processes or the assessment of a status quo in the runup of a treatment, a measurement of a single focal plane image (*xy*) at a single time point might be fully sufficient to detect an abnormality, such as a tumor (Figure 1A). 3D representation (*xyz*) can further assist in capturing the spatial dimensions of a feature of interest (Figure 1B). However, when estimations on the time scale of changes or investigations on an underlying mechanism are required, increasing the sampling density in the temporal domain extends the dimensionality of the measured data to the 3D (*xyt*) or even 4D (*xyzt*) space (Figure 1C,D). Depending on the speed of dynamics of the investigated samples and the ability to halt the process at a certain state for later analysis, two sampling approaches come into hand. The first one includes a repeated sample extraction on set time points from the same or different donors while the second one concerns a semicontinuous *ex*- or *in vivo* observation of a live process. The key advantage of the latter methods lies in their information content, notably the elimination of intersample variability in a time series of recorded data. Thus, directly linking observations within a series to their temporal course becomes possible within the approach of (semi)continuous observation and theoretically enables a number-based evaluation of features to assess the temporal development qualitatively and quantitatively. The term “semicontinuous” thereby indicates that the temporal sampling density is limited by the acquisition of the spatial domain and thus is strongly dependent on spatial resolution, scan speed, and image quality. While research merely focuses on the improvement of spatial and temporal resolution,^4−6^ an implementation of 4D (*xyzt*) imaging using nonlinear multiphoton techniques for biomedical tissue analysis remains relatively limited,^4−7^ particularly in the context of feature extraction.^6^ Critical features can include dimensional data of the sample or important compartments, as well as method-specific parameters such as intensities or spectral shifts. Here we aim to explore how a 4D-spatiotemporal sampling approach using a confocal laser scanning microscope and temporal resolutions in the minute range can assist in an understanding of dynamic processes. Specifically, we focus on the drug-induced effects on dura mater thin sections to extract and evaluate critical features.

**Figure 1:**
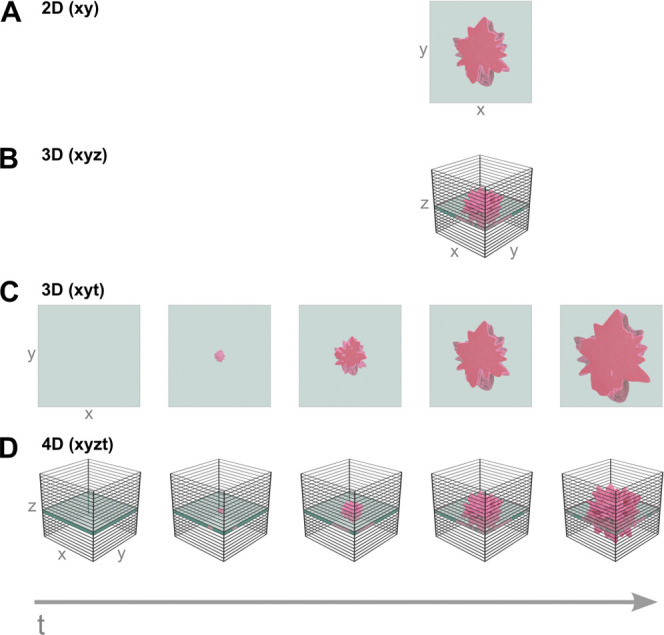
Illustrative scheme depicting the dimensionality of imaging in capturing dynamic processes (here the growth of an artifact), progressing from single-point 2D imaging to spatiotemporal 4D imaging. The areas shown in panels (A) and (C) correspond to the highlighted layers in panels (B) and (D), respectively.

The dura mater is the outermost and thickest of the three meningeal layers that protectively surround the brain and spinal cord.^8^ Structurally, it consists mainly of elastin and collagen fibers, forming a dense and tough network in healthy dura mater tissue.^8^ Throughout the dura, the fiber network is not homogeneous, but rather made of different layers parallel to the outer surface that differ in fiber alignment and density,^8,9^ eventually shaping the biophysical and mechanical properties.^10,11^ Given these aspects, any damage to the dura mater or its filigree collagen structure should be urgently prevented.

Within the clinical sector, an emerging research interest arose in the last decades for the prevention and management of dural damages, mainly due to mechanical causes (e.g., tearing or penetration during spinal surgery).^12,13^ In contrast, there is limited information available regarding substance-induced alterations in dura mater tissue, although the dura is before and during spinal surgery necessarily in close contact with a variety of drugs. Diffusion of drugs through the dura mater tissue is, for example, essential for transport into and distribution in the cerebrospinal fluid (CFS) after epidural administration. Additionally, prevention and treatment of postoperation complications, such as surgery site infections (SSIs), might elicit ancillary drug administration. Apart from surgical revision, nowadays the topical administration of antibiotics and local antiseptic treatments to eliminate bacteria have emerged as a powerful intraoperative prevention strategy as they can significantly reduce the incidence of SSIs after spinal surgery.^14−16^ As in the topical administrations of e.g. antibiotics the locally applied concentration can easily exceed the concentrations usually desired (e.g., minimal inhibitory concentration),^17^ we anticipate that toxic side effects on local and surrounding tissue might not be ruled out completely. It was previously shown, that high concentrations of NaOCl, the reactive agent of LAVANOX-Serag (0.08% sodium hypochlorite), have indeed the potential to damage collagen frameworks.^18,19^ However, there is still a lack of evidence on the toxicity of the drug formulations themselves on collagenous tissue and more specifically on dura mater.^20^

From a microscopic point of view, we assume that any drug-induced variations of the collagen framework may eventually lead to severe tissue damage and an unwanted penetration rate of drugs into the CSF. While the resulting effects may be similar, significant differences in the damaging patterns of the collagen framework and/or the dynamics could be present. To illustrate, it is well known in the literature that heat or specific enzymes such as e.g. collagenases can damage collagen frameworks.^21,22^ While collagenases are typically designed to cleave after a particular amino acid sequence and thus break down the strands into subunits, heat rather denatures collagen by an unwinding of its helical structure, which then results in gelatinization. For this reason, we anticipate observing drug-induced changes in the dura mater tissue as a variable pattern of damage to collagen. Among others, those changes may thus include the release of small amino acid fragments, the crimping of the tissue by structural rearrangements, and the loosening of rigidity by, e.g., swelling processes. All of these processes may eventually manifest as changes in dimensional properties such as width, height, and overall volume on a microscopic scale.

Type I collagen is an ideal target for nonlinear imaging using second harmonic generation (SHG), as already reported for the study of fibrillar collagen in various tissues.^10,23−26^ Illumination of a sample that acts as a so-called χ^(2)^ medium by a strong oscillating electromagnetic field of frequency *ν* and wavelength λ can lead to the emission of light of frequency 2*ν*, which corresponds to a halving of the initial excitation wavelength. Due to the underlying physical principles, SHG does not occur in every material but only in noncentrosymmetric media and at sites of broken symmetry (e.g., at surfaces). Type I collagen as present in the dura mater fulfills this condition due to its helical shape in its primary and secondary structures, leading to an overall anisotropy. Another effect that favors type I collagen for SHG imaging is the hierarchic and highly ordered structure, which leads to a strong SHG signal by constructive interference of the signal by adjacent emitters.^27^

The high abundance of collagen in the dura mater as well as its good trackability by nonlinear techniques (particularly SHG) make type I collagen ideal for the evaluation of drug-induced structural changes in the dura mater by microscopic means. We claim that spatiotemporal SHG imaging of dura mater thin sections after drug administration enables us to reveal structural and dimensional changes caused by mechanical as well as chemical origins. Therefore, we applied a selection of drugs in commercially available concentrations that are typically used during spinal surgeries. The medications included antibiotics, antiseptics, antifibrinolytics, local anesthetics, and water and collagenase solutions as control agents.

We identified highly concentrated vancomycin solutions and the LAVANOX-Serag-formulation as potentially damaging, inducing a distinct swelling of the thin section in width associated with an overall loss of SHG signal.

## Experimental Section

### Sample Preparation

For the study of nondiffusion-limited effects by simultaneous application of drugs to all dural layers, 30 μm thick tissue sections were cut perpendicular to the dura surface with a cryotome at −20 °C. With all tissue samples originating from parallel sections of the same bulk piece of a sheep’s spinal dura mater, we sought to minimize the variation in the native state from specimen to specimen as much as possible. The thin sections were put on glass-bottom dishes and were immediately stored at −80 °C until further use. Before the drug treatment, both ends of the frozen thin section were fixed with UV-curing glue, leaving only a few millimeters large window uncovered to prevent floating of the thin section in the treatment solution. The remaining space was wet with a drop of drug solution, ensuring enough reservoir to have the thin section embedded in the liquid during the whole measurement process. The addition of the drug solution marked the beginning of treatment (*t* = 0). Due to the thin width, the section immediately defrosted after drug addition and was transferred to the microscope. The first stack could be recorded between 2 and 5 min after drug addition, which is the time span that was necessary for sample mounting and measurement setup. A schematic of the workflow is provided in the Supporting Information (Figure S1).

### SHG Measurements

The spatiotemporal SHG measurements were performed with an inverse confocal laser scanning microscope (Zeiss 510 Meta, Zeiss, Germany) with incoupling of external lasers as described earlier.^28^ Light emitted from a diode-pumped Nd-vanadate laser (532 nm, Verdi-V18, Coherent, USA) was used as the pumping source for a Ti:sapphire laser (Mira, Coherent, USA) generating femtosecond pulses at 830(10) nm (100 fs, 76 MHz) which were used for SHG generation. The beam was guided into the microscope and focused onto the sample (≈67 mW at the sample) by a 20×/0.8 objective (plan-apochromat, Olympus, Japan). The SHG signal emitted from the sample was separated by a set of suitable optical filters (forward: SHG415 + 650sp, backward: 650sp (Semrock) + 415/3 (Omega Optical, USA)) and registered in forward (f) and backward (epi) directions by photomultiplier tubes (R6257, Hamamatsu Photonics, Japan). While the signal in the epi-direction was recollected by the objective, a condenser (0.8 NA) was used for the collection of signal in the forward direction.

To obtain spatiotemporal data, a time series of *z*-stacks was started right after the drug addition. Ensuring that the thin section volume remained within the imaging volume throughout the entire time span was critical for accurate data assessment during processing following the image acquirement.

While the thin section samples were already stabilized by gluing spots in the *y*-direction (see Figure S1), swelling or floating processes were particularly found to influence the thin section’s position within the imaged volume over time in the other two dimensions. To address these issues, a safety margin was added to the cut section width when determining the necessary *z*-dimensions for the stack. Typically, stacks containing a minimum of 61 slices with a spacing of 1 μm were sufficient for most of the samples. Samples, where the thin section left the imaging volume in either the *x*- or *z*-direction during the imaged time span, were omitted from further analysis.

For image acquisition, the following settings were used for each slice: objective: 20×/0.8 NA plan-apochromat, zoom: 1.0, field of view: 512 px × 512 px, 450 μm × 450 μm, pixel dwell time: 1.60 μs/px, averaging: 2 lines (mean).

### Extraction of Features from the 4D-Stacks

All data series were cropped to an upper border of at maximum 50 min for consistency reasons. For a detailed description of the procedures, refer to the Supporting Information. The used *ImageJ* and *Python* scripts are also provided as a part of the Supporting Information.

## Results and Discussion

**Figure 2:**
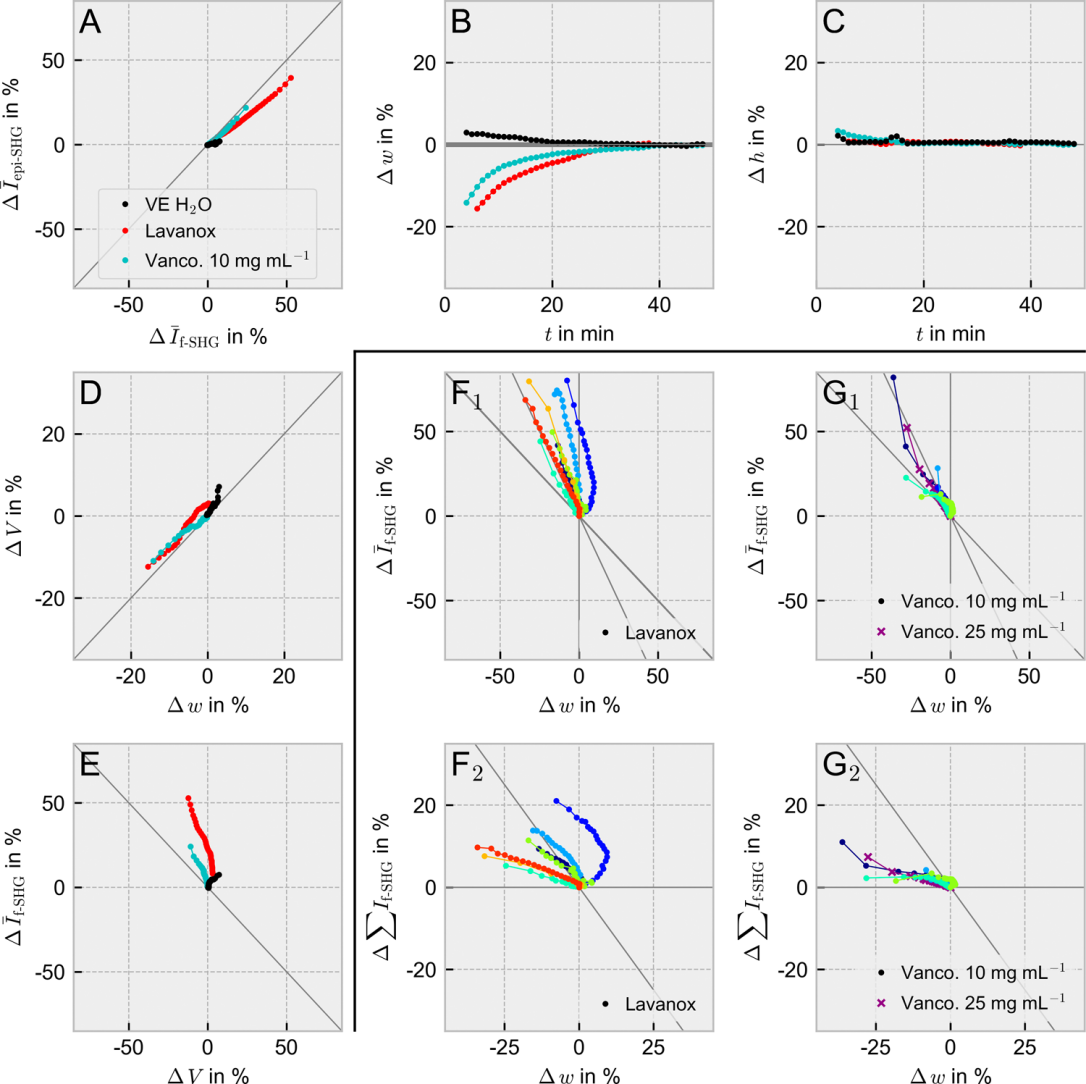
Selected feature plots for behavior description. (A–E) compare changes in dimensional properties (volume *V*, width *w*, height *h*) and the recorded mean SHG signal within the sample volume (*I̅*) in forward (f-) and backward (epi-) direction. Shown are the mean curves of all individual samples for VE H_2_O (black), 10 mg mL^–1^ vancomycin hydrochloride solution (cyan, Vanco.), and the commercially available LAVANOX-Serag solution (red, Lavanox). (F, G) provide 2D-correlations between the increase in width and the loss of the average (*I̅*, F_1_, and G_1_) or cumulative (∑*I*, F_2_, and G_2_) SHG signal in the segmented sample volume for LAVANOX-Serag (F) and vancomycin hydrochloride (G) solutions. Individual samples were visualized with different colors. For vancomycin hydrochloride, we additionally provide a curve for a 25 mg mL^–1^ solution (purple) which exhibits a similar behavior to the 10 mg mL^–1^ solutions that are the focus of this figure. However, we point out that replicates were only measured for the 10 mg mL^–1^ solution. Gray lines serve as optical guidance.

Determining the harm potential of a drug in clinical application is complex and depends on a variety of factors, including the aggressiveness (e.g., pH and reactivity) of the drug, the concentration used, and the duration of the interaction, the diffusion constant in tissue and biological half-life, as well as patient-specific characteristics such as age or the condition of the dura itself. Nevertheless, *ex vivo* studies in model systems at least allow for the identification of candidates that show a likelihood of damage, thereby narrowing down the broad spectrum of available agents to the essence of interest that should be placed in the focus for subsequent clinical patient studies. Here, we investigated diffusion-unlimited changes on the entire dura layer system as a worst-case scenario using 30 μm thin sections of the same dura mater piece that were cut perpendicular to their outer surface. As reuse of the identical sample for multiple experiments was impossible, we consider the choice for sections from the same bulk of a single donor as fair. In terms of comparability of drug results the variations between sections of the identical dura piece will be likely less pronounced than between individual donors.^11^ For elucidating the dynamics of changes in dura mater tissue, we implemented a 4D-spatiotemporal SHG imaging approach, revealing information on the kind and order of changes and their potential origin. A detailed collection of feature plots extracted from the SHG data and a sample-to-sample evaluation are provided as a part of the Supporting Information (Figures S2–S15). Figure 2 distills the most essential findings from the comprehensive supporting figures by providing summarizing plots for vancomycin hydrochloride (10 mg mL^–1^), LAVANOX-Serag, and demineralized water (VE H_2_O) which we have identified as key substances for behavior description and replicated multiple times. In the following, we will systematically discuss the embedded information on the SHG signal using Figure 2 as the structural framework.

The most easily accessible biomarker for registering changes to the collagen framework is the development of the mean SHG intensity with interaction time in the thin section volume that can be detected in either the forward (f-SHG) or backward direction (epi-SHG) for biological tissues (Figures S5 and S6). The ratio of forward to backward-emitted intensity thereby relies strongly on the phase matching condition and was previously found to depend e.g., on the randomization of fibril orientation and fibril size.^29^ However, back-reflection of the forward-emitted signal as well as absorption processes within tissue or surrounding medium can significantly alter the expected forward-to-backward ratio. For the samples investigated herein, the SHG signal emitted in the forward direction was more intense with an exception for the dark collagenase solution. Nevertheless, in any case, both signals proved to be equally suited, showing the same qualitative behavior as the 2D correlation plot between f-SHG and epi-SHG signal intensities revealed (Figures 2A, S5, S6, and S9). While for a fixed linear polarization of the exciting laser, the recorded SHG intensity in general will depend on the fiber direction which might result in varying absolute values for different dura orientations.^30^ As we did not alter the sample position during a time series, consistency between the SHG values within a series is ensured. To minimize systematic deviations between samples, it was additionally confirmed that all samples were oriented along the same axis, with the outer dura surface always pointing to the same side.

The most severe percentual losses from the forward and backward-emitted intensity, respectively, with respect to time in the sample volume were particularly observed for collagenase solution, LAVANOX-Serag, and vancomycin hydrochloride >10 mg mL^–1^ whereas the SHG intensity of other wound irrigation solutions, tranexamic acid, Xylocitin-loc, and the negative control VE H_2_O rather showed a stagnant or only slightly decreasing behavior (Figures S5 and S6), Particularly for collagenase solution and all LAVANOX-Serag samples, the drop in signal is very pronounced, although individual positions and curve shapes may vary for replicates of LAVANOX-Serag. Decreasing SHG intensity in general indicates a reaction of the tissue section to the drug environment. However, implications on the specific tissue reaction, e.g., swelling, primary structure destruction, or others, require additionally a consideration of changes in dimensional properties and cannot be drawn solely from the development of SHG signal with time.

**Figure 3:**
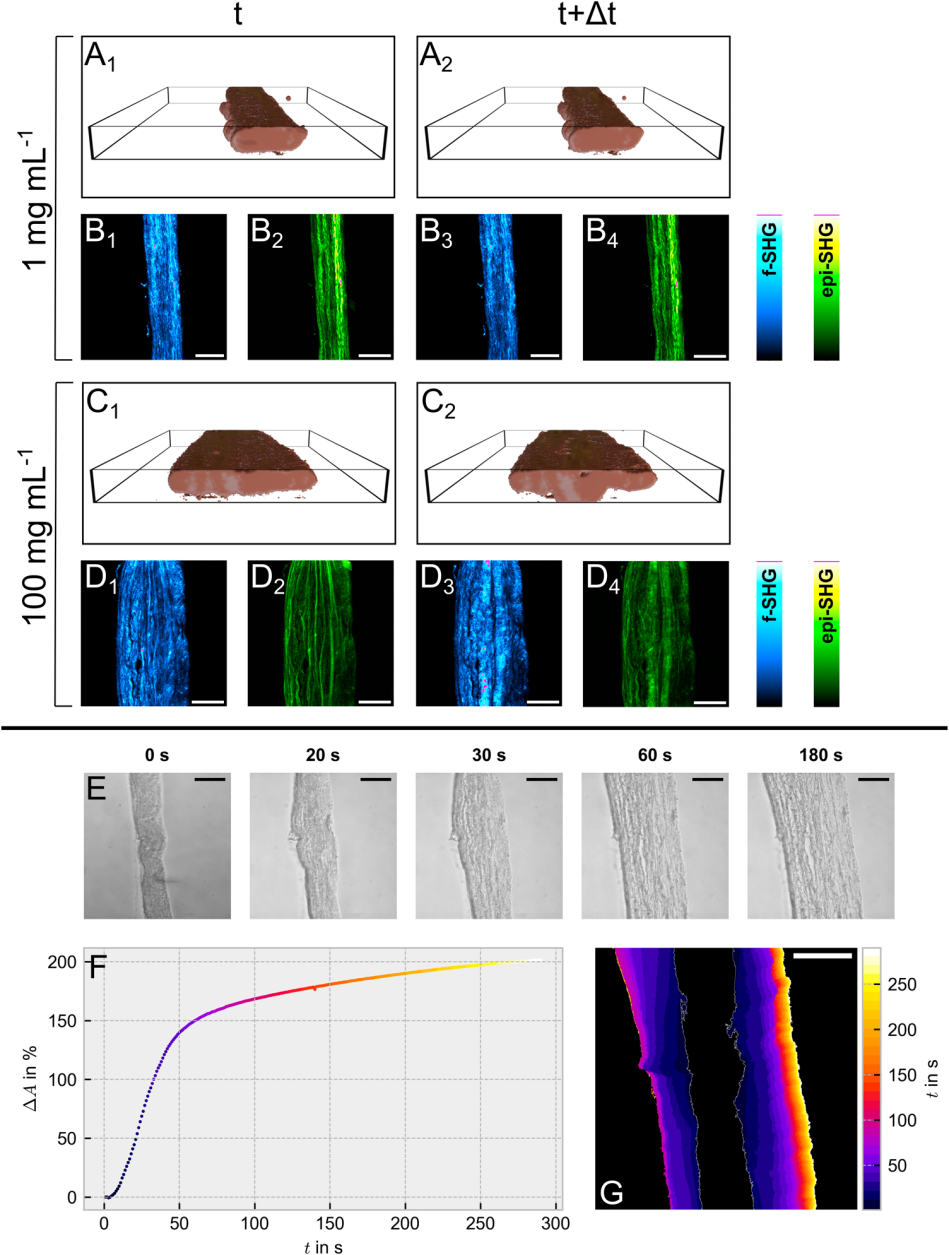
Analysis of the swelling behavior of sheep’s dura mater treated with differently concentrated vancomycin solutions. (A–D) show the results obtained for 4D-SHG imaging of select samples treated with 1 mg mL^–1^ (A, B) and 100 mg mL^–1^ (C, D) vancomycin hydrochloride in water. Shown are the volume reconstruction (A, C) of the samples at the beginning (A_1_: 2.5 min, C_1_: 2.3 min) and after approximately 30 min (A_2_: 32.7 min, C_2_: 32.5 min) and the corresponding f-SHG (B, D, blue) or epi-SHG (B, D, green) sum projections of the measured stacks. For the SHG images, the indices 1 and 2 correspond to the earlier time point, whereas 3 and 4 denote the later time point. The black boxes in (A) and (C) indicate the imaged volume. (E–G) depict the swelling of a sheep’s spinal dura mater thin section treated with a 100 mg mL^–1^ vancomycin hydrochloride solution by means of light microscopy. (E) shows light microscopy photographs for selected times after the drug addition. (F) The sample swelling within the 100 mg mL^–1^ solution in the first seconds after drug addition was calculated from the area in the light microscopic images (sampling density: 1 image per second) that was covered by the thin section. The classification of the sample and background was performed using the trainable WEKA plugin^31^ in *ImageJ*. (G) provides a color-coded visualization of inhomogeneous sample swelling in the WEKA-classified maps in intervals of 10 s after the addition of the 100 mg mL^–1^ vancomycin solution. The original sample width was indicated by a white margin. Scale bar size in all images: 100 μm.

Besides coping simultaneously with sample motion in the imaging volume, the main advantage of 4D-spatiotemporal imaging over imaging in a single focal plane is the ability of tissue reconstruction (Figure 3A,C), which perfectly suits the aforementioned demands. Tissue reconstructions from microscopic *z*-stacks using proprietary software are well established, but open source, and thus widely available, options are lacking and suffer from several problems that exacerbate finding settings that are transferable between samples and allow for a scientifically sound result. Among others, challenges particularly arise in defining tissue boundaries. These include differentiating between the actual low signal in outer layers (particularly in the *z*-direction) and contributions from out-of-focus layers as well as from noise. Additionally, the treatment of holes within the sample and their distinction from areas of a low SHG signal are crucial. The aforementioned decisions proved even more complex when considering the varying overall SHG intensity with time due to a substance-sample interaction. In terms of dimension retrieval, the assessment of a sound voxel-based joint tissue area and the calculation of volume, mean width, and mean height in highly tilted samples within the *x*–*z*, *y*–*z*, and *x*–*y*-plane as well as various combinations thereof demanded sophisticated processing. All geometric features were eventually reliably calculated from the f-SHG signal using exclusively open-source software. For details on the calculation procedure please refer to the Supporting Information (Figures S17–S21). Our evaluation approach also proved very robust against the recorded *z*-stack depths and thus the sampling density in the time domain, as the volume segmentation for the VE H_2_O samples revealed. Due to a more severe tilt, one of the three VE H_2_O samples required a stack depth of 91 slices instead of 61 to cover the entire sample. As an increase in stack depth leads to a longer measurement time per stack, assuming that the pixel dwell time and single slice dimensions remain unchanged, the time points being probed become less dense when stacks are measured continuously. Nonetheless, all three replicates coincided nicely with the percentual changes in volume, height, and width as long as the sample does not leave the set imaging volume during the investigated period. For this reason, we assume that any observed changes compared to the behavior of the VE H_2_O control samples can thus be directly attributed to the influence of the applied drug formulation under study for non-negative control samples.

Thanks to the ability of tissue reconstruction, deep insights into dimensional properties and their correlation along the so-far evaluated average SHG signal are feasible. Concerning the geometric features, three outcomes became visible: First, changes in width are again predominantly observed for LAVANOX-Serag, vancomycin hydrochloride, and collagenase solution and express themselves by a swelling of the tissue section with time (Figures 2B and S2). Second, except for the collagenase solution, the height is unaffected^a^ by the swelling process (Figures 2C and S3) which will lead in all other cases, depending on the width development, to a constant or increasing volume over time (Figures 2D, S4, S10, and S11). And third, a concentration dependence becomes present for vancomycin hydrochloride solutions. For concentrations between 1 and 25 mg mL^–1^, the swelling is trackable by SHG imaging (Figures S2–S15), whereas for the highest concentration of vancomycin hydrochloride (100 mg mL^–1^), the registered curve is flat, both in terms of volume or width (Figures S2–S4). Based on the large sample width (Figure 3C,D vs Figure 3A,B), it was hypothesized that the swelling took already place in a time shorter than the sample mounting and measurement setup time, which was verified by light microscopy revealing almost a doubling in area in the first 30 s after drug addition (Figure 3E–G).

Another benefit of 4D (*xyzt*) imaging is the ability to connect different features to conclude their progression. 2D-correlations between percentual changes in dimensions of volume, height, and width (the length dimension was fixed) and the SHG signal further enabled an educated guess on the interaction type and the isotropy character of swelling processes, if present. We thereby envisioned two scenarios that can influence the SHG signal in two distinct ways. Clipping within the amino acid sequences or unwinding of the collagen fibrils can result in disruption of the collagen morphology. Any of the mentioned causes can, thus, lead to a reduction in both the average and cumulative SHG signal in the sample volume. Loosening of the collagen architecture might eventually cause structural weakness, which might express itself in a swelling of the tissue section. On the other hand, the weakening of the interactions between the individual fiber bundles is probably the cause of severe swelling associated with the appearance of holes in the tissue and the drifting apart of larger tissue fractions. In that described case, we expect a different behavior, namely, a decrease in the average SHG signal per sample volume, but no large variations in the cumulative SHG numbers. Our hypothesis was supported by the collagenase solution, which showed a more intense decrease in the average SHG intensity as it was observed for the increase in width (Figures S12 and S13). Additionally, the summed SHG signal per volume decreased during sample swelling (Figures S7 and S8). Following the above arguments, both feature plot series (Figures S5–S9 and S12–S15) thus indicate the alteration of the internal structure of the collagen fibril rather than a loosening of rigidity on a more macroscopic scale. The drawn conclusions match very well given that collagenases are designed to clip after certain amino acid sequences. Between the two drugs in question, LAVANOX-Serag and vancomycin hydrochloride, the origin of the change seems to be different. Whereas the loss in the average SHG signal for LAVANOX-Serag is steep (Figure 2E,F_1_) and resembles the drop for collagenase (Figures S12 and S13), vancomycin hydrochloride shows a decrease in the average SHG signal (Figure 2E,G_1_) that mainly correlates with the broadening in width. The evaluation of the summed SHG signal in the sample volume showed a large range of variations for LAVANOX-Serag but an almost steady behavior for vancomycin hydrochloride (Figures 2F_2_,G_2_, S7, and S8). Consequently, we conclude that for LAVANOX-Serag, the interaction of the drug and thin section mainly affects the intrinsic structure of the collagen framework. Vancomycin hydrochloride, however, predominantly shows swelling caused by the drifting of larger bundle frameworks. No decrease in SHG signal for solutions of different osmolarity (VE H_2_O, PBS buffer solution, and sat. NaCl solution, data not shown) was observed for approximately 50 min long treatments.

From a chemical point of view, we did not expect reactions from the core vancomycin structure with collagen. For this reason, we conclude that changes to the dura mater tissue can thus be mainly attributed to the characteristics of the formulation. Specifically, we determined the pH of all tested solutions (Table S1). Deviations from the modal pH values of the screened solutions were observed particularly for LAVANOX-Serag and the higher concentrated vancomycin hydrochloride solutions. For vancomycin hydrochloride solutions, effective swelling and SHG loss were observed for the solutions >10 mg mL^–1^ which all show a pH lower than 4 (Figure S16), whereas for the 1 mg mL^–1^ solution, the effects were not well pronounced (Figure 3A_1,2_,B_1–4_). The effect of pH on artificially made collagen complexes and extracted or isolated fibrils is well-studied.^32,33^ Swelling manifests itself specifically in acidic pH values, resulting in an increase in weight due to repellant forces that promote assembly in more porous structures with internal voids. However, evidence for the swelling of complexly composed native collagenous tissues is only given for selected examples^34,35^ and in particular not for the influence of drug-induced pH environments. Nonetheless, investigations on rat tendon^34^ support our observations suggesting a splitting up of collagen bundles in acid pH into fibers and fibrils as concluded from our SHG measurements as well.

For LAVANOX-Serag, several explanations seem feasible and are in good agreement with our conclusion of mainly primary and secondary structure destruction. Concerning LAVANOX-Serag’s reactive agent NaOCl, it was previously shown that hypochlorite has the potential to dissolve dentin collagen^19^ as well damaging collagen in organic matter^18^ for concentrations >1% (*m*/*v*). It was thereby found that the damage correlates with the presence of OCl^–^ and thus becomes important for high concentrations and low pH values.^36,37^ However, the concentration of hypochlorite in LAVANOX-Serag is quite low at <0.08% OCl^–^ from NaOCl/HOCl with a pH of ∼8.5. Although the concentration dependence may vary significantly between diffusion-limited and nonlimited scenarios, it seems unlikely that the interaction of hypochlorite with the dura is the main source of damage. Similar to our conclusions for vancomycin, it was rather anticipated that the pronounced damage to the thin dura section might be attributed to the formulation’s pH, which is high (∼8.5) compared to the other tested drugs (Table S1). Analogous to observations in acidic environments, the deprotonation of functional groups under alkaline conditions can induce repellant forces between strands, eventually resulting in swelling of collagen.^33^ Moreover, investigations on collagen in fish skin revealed significant morphology changes of collagen in electron microscopy with increasing pH.^38^ Within the LAVANOX-Serag-formulation’s characteristic pH range, in particular, a weakening of the collagen’s sheet-like structure was observed which was grounded on changes in protein folding on the secondary structure level. When the pH increases, the prevalence of β-turns and random coils intensifies which results in collagen unfolding and, therefore, to an overall looser structure.^38^ The damage to the collagen hierarchy by the weakening of the secondary structure matches very well with the conclusions drawn from 4D-spatiotemporal SHG imaging presented here.

## Conclusions

SHG-related imaging featuring the possibility of sample reconstruction proved to be a viable imaging technique for the evaluation of temporal changes in collagenous tissues. For the scenario studied herein of investigating drug-induced changes to dura mater tissue, it was found that topical administration of drugs on dura mater thin sections can cause structural disruptions of the collagen architecture. Through spatiotemporal imaging, an assessment of dimensional properties over the course of time became possible. A correlation of changes in dimensional properties with the temporal decrease of SHG signal eventually allowed for an evaluation of the origin of damage that cannot be drawn from single focal plane imaging. Among the probed medications, LAVANOX-Serag and vancomycin hydrochloride solution did induce changes to the dura mater tissue. We eventually linked the observed disruption patterns of the collagen framework to the pH of the drug formulations and concluded that vancomycin hydrochloride mainly affects interbundle interactions, while LAVANOX-Serag likely damages collagen on the secondary structure level. Consequently, both interactions lead to a profound swelling of the thin section volume and width as accessed by tissue reconstruction. The precise linkage of pH, swelling, and protein folding or cleavage might elicit subsequent studies.

We are aware that the study design limits direct implications to in-patient behavior. This concerns, in particular, the amount of swelling in *in vivo* experiments as well as the reversibility of the swelling and caused body reactions, which need to be the focus of clinical studies. At present, the extent to which swelling in diffusion-limited scenarios (such as bulk tissue) compares to the nondiffusion-limited cases studied here remains uncertain. Within the body, buffering and dilution effects during prolonged incubation can take place, which potentially alters the effective pH of the drugs tested here. While we do not expect such effects to become critical for the *ex vivo* scenario studied here, we emphasize the need for verification in clinical *in vivo* experiments. As previous studies have shown, swelling in response to pH seems to be kind of reversible for collagen fibers but may become irreversible for mixed complexes.^33^ The specific implications of potential reversibility of swelling and whether such processes occur within the body could provide a valuable starting point for further research in the clinical domain. Nonetheless, from a spectroscopic point of view, we implemented a simple measurement strategy that enabled the tracking of drug-induced changes in the collagenous tissue. We further envision 4D-spatiotemporal SHG imaging as feasible for screenings of a multitude of collagenous tissues, such as, for example, tendon, skin, cornea, and cartilage as well as various disruptive factors such as, for example, temperature, salinity, concentration, or presence of reactants which also might have different influence on different tissue types.

During tissue reconstruction, we found that swelling showed a pronounced anisotropy. Precisely, we observed a swelling in width, while the height of the thin section remained unaffected. However, we also noticed that the swelling on the left and right sides of the thin section was inhomogeneous (Figure 3G). Since thin sections were cut perpendicular to the outer dura surface, we hypothesized that the depth-dependent differences in the general collagen architecture might be a contributing factor.

## Supporting Information Available

Several comprehensive feature plots and more detailed descriptions on the extraction of dimensional properties such as width, height, and volume (PDF)
Used scripts and the WEKA classifier. (ZIP)

## Footnotes

a For the LAVANOX-Serag samples, we certainly observed a spread within the curves. However, it needs to be mentioned in that regard, that the error in height is comparably large in terms of the determined sample height, which is not the case for the sample width. For a cut sample height of 30 μm and a *z*-spacing of 1 μm between slides, two pixels misassigned each at the top and bottom boundary of the thin section during the segmentation procedure will make up an error of 13%. Thus, the observed variation of <20% is not as severe as it appears at first sight.
