# Supplementary material for: 4D-Spatiotemporal SHG Imaging for the Analysis of Drug-Induced Changes in the Dura Mater
Source: Anal Chem. 2025 Feb 14;97(7):3892–900. doi: 10.1021/acs.analchem.4c04887 (PMC11866281; doi:10.1021/acs.analchem.4c04887)
Supplement: Supplementary file 1 — ac4c04887_si_001.pdf [file ac4c04887_si_001.pdf]

Supporting Information

4D-spatio-temporal SHG imaging for the analysis of drug-induced changes in dura mater

Constanze Schultz[1], Marko Rodewald[1,†], Andreas Weidisch[2], Tobias Meyer-Zedler[1,3], Thomas Caffard[4], Michael Schmitt[3], Georg Matziolis[2], Timo Zippelius[4,#], Jürgen Popp[1,3,5]

[1]Leibniz Institute of Photonic Technology (Leibniz-IPHT), Member of Leibniz Health Technologies, Member of the Leibniz Center for Photonics in Infection Research (LPI), Albert-Einstein-Str. 9, 07745 Jena, Germany

[2]Orthopedic Department, Jena University Hospital, Campus Eisenberg, Klosterlausnitzer Straße 81, 07607 Eisenberg, Germany

[3]Institute of Physical Chemistry (IPC) and Abbe Center of Photonics (ACP), Member of the Leibniz Center for Photonics in Infection Research (LPI), Friedrich Schiller University Jena, Helmholtzweg 4, 07743 Jena, Germany

[4]Department of Orthopedic Surgery, University of Ulm, Oberer Eselsberg 45, 89081 Ulm, Germany.

[5]Cluster of Excellence Balance of the Microverse, Friedrich Schiller University Jena, Fürstengraben 1, 07743 Jena, Germany

† Present address of M. R.: Dipartimento di Fisica, Politecnico di Milano, Piazza Leonardo da Vinci 32, 20133 Milan, Italy
# Present address of T. Z.: Spine Surgery, Orthopedics, Traumatology, SRH Klinikum Karlsbad, Guttmannstraße 1, 76307 Karlsbad-Langensteinbach, Germany

Contents

1 Supplementary Data 2
1.1 Change of features over the course of time 3
1.2 2D-correlation maps between different features 6
1.3 pH of the tested solutions 10
2 Computational analysis of dimensional properties based on the forward-emitted SHG signal 11
2.1 Determination of the brightest plane 11
2.2 Determination of the average dura width 13
2.3 Volume reconstruction 15
2.4 Determination of the average dura thickness 16
3 References 17

1 Supplementary Data

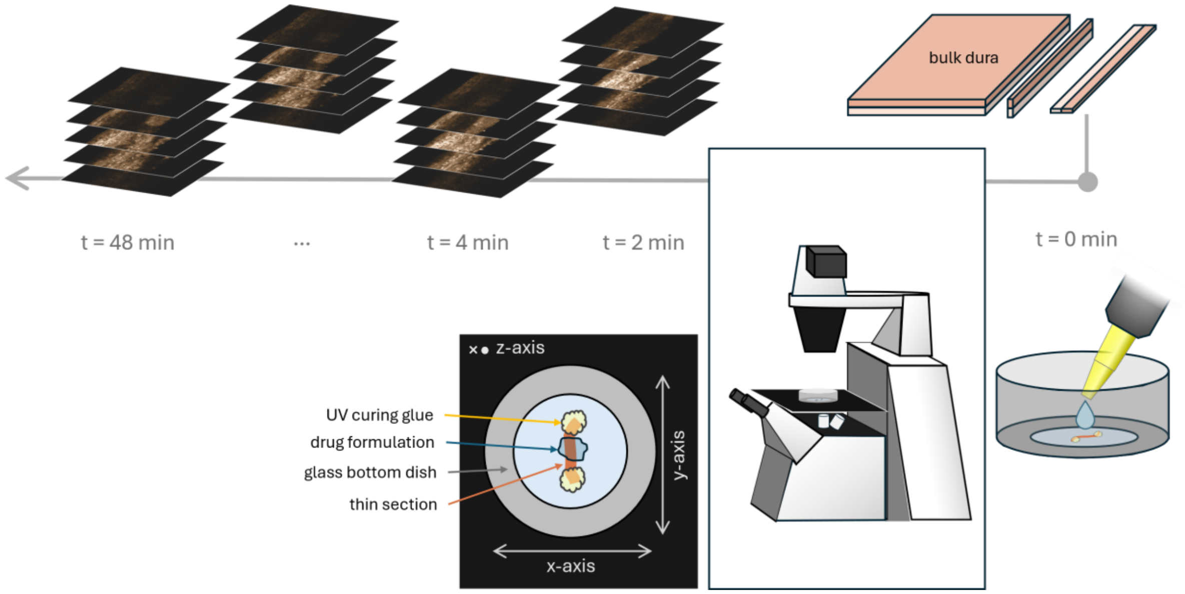

Figure S 1: Workflow for the spatio-temporal SHG measurements. Bulk dura pieces were cut into thin sections perpendicular to the dura surface and glued on glass bottom dishes. The schematic of the mounted sample shows the assignment of the axis as used in this paper. After the addition of the drug formulation z-stacks were measured to get insights into temporal changes of the thin sections.

1.1 Change of features over the course of time

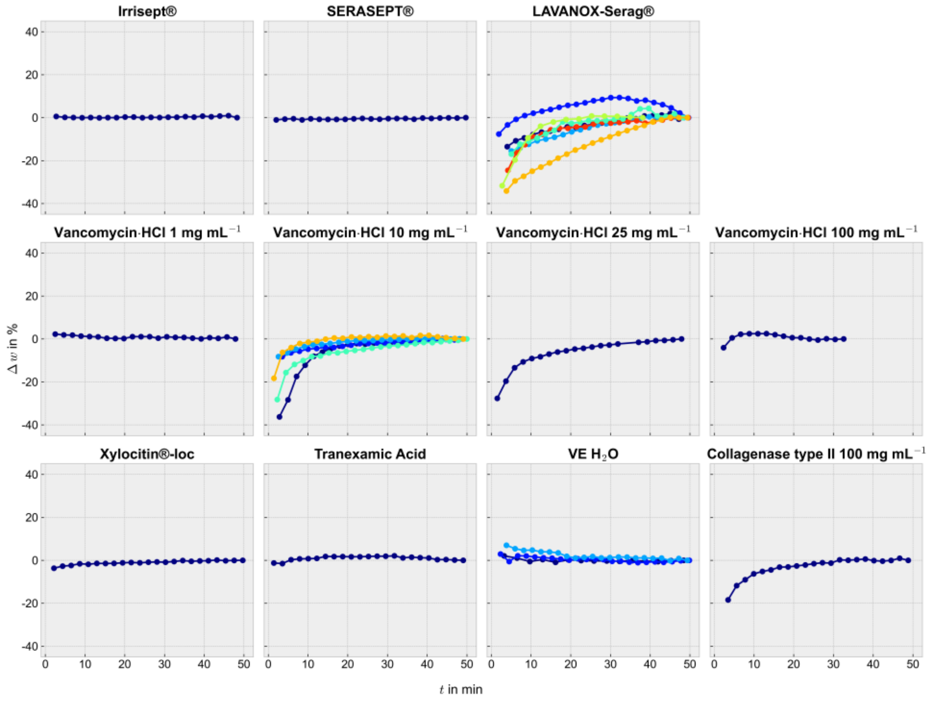

Figure S 2: Change of the thin section width of the dura mater section with respect to time after the addition of medications/media. For better visualization, the last measured point was used as a reference (0-line) and percentual changes were calculated with respect to this value. For more information on data evaluation, please refer to the main text.

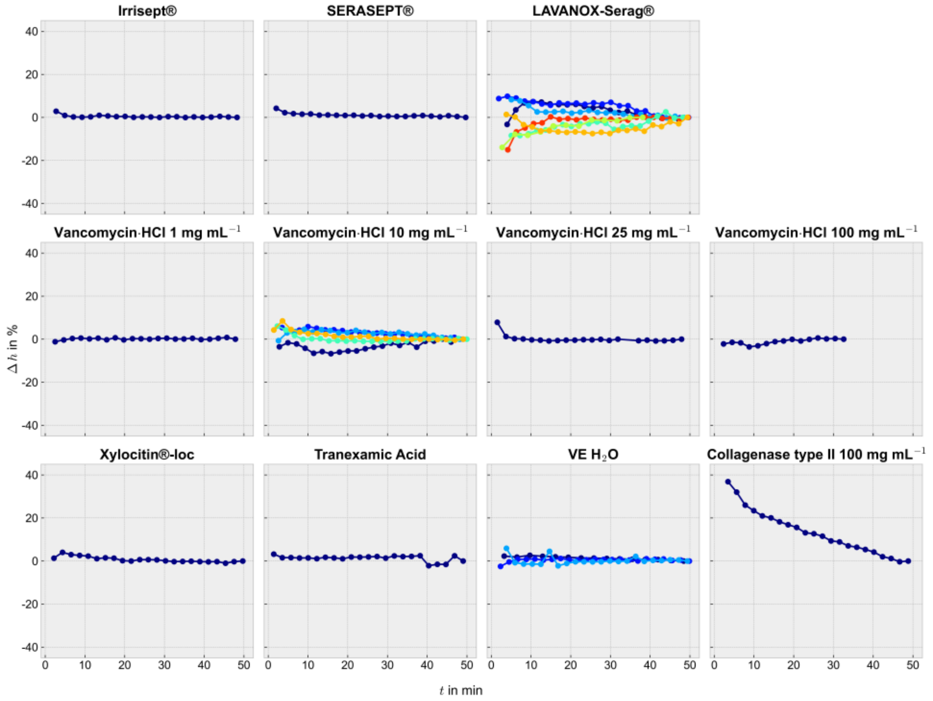

Figure S 3: Change of the thin section height of the dura mater section with respect to time after the addition of medications/media. For better visualization, the last measured point was used as a reference (0-line) and percentual changes were calculated with respect to this value. For more information on data evaluation, please refer to the main text.

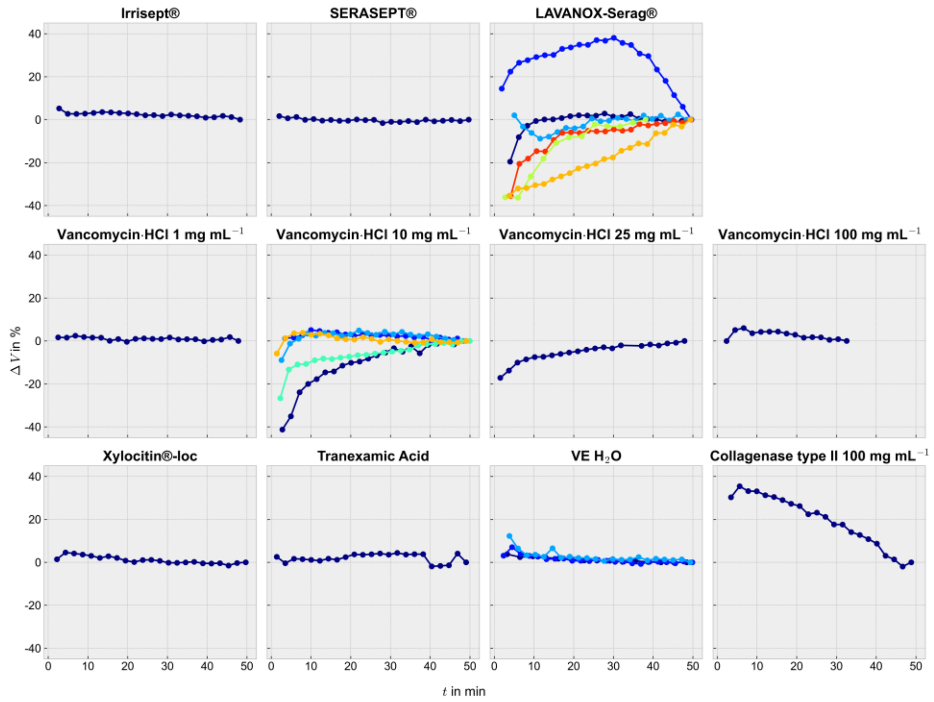

Figure S 4: Change of the thin section volume of the dura mater section with respect to time after the addition of medications/media. For better visualization, the last measured point was used as a reference (0-line) and percentual changes were calculated with respect to this value. For more information on data evaluation, please refer to the main text.

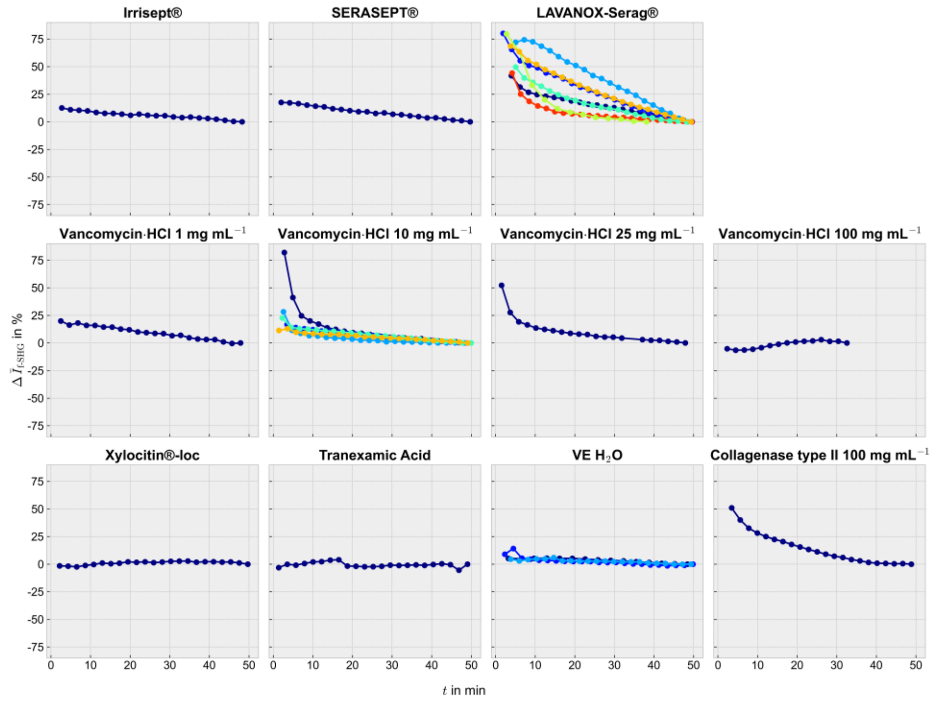

Figure S 5: Change of the average f-SHG signal in the thin section volume of the dura mater section with respect to time after the addition of medications/media. For better visualization, the last measured point was used as a reference (0-line) and percentual changes were calculated with respect to this value. For more information on data evaluation, please refer to the main text.

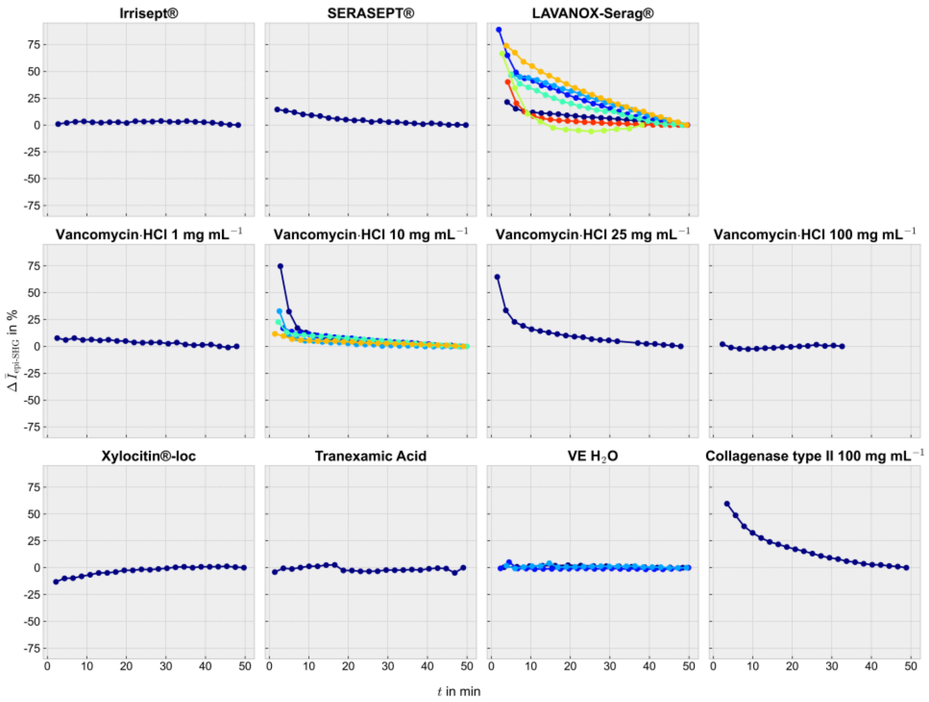

Figure S 6 Change of the average epi-SHG signal in the thin section volume of the dura mater section with respect to time after the addition of medications/media. For better visualization, the last measured point was used as a reference (0-line) and percentual changes were calculated with respect to this value. For more information on data evaluation, please refer to the main text.

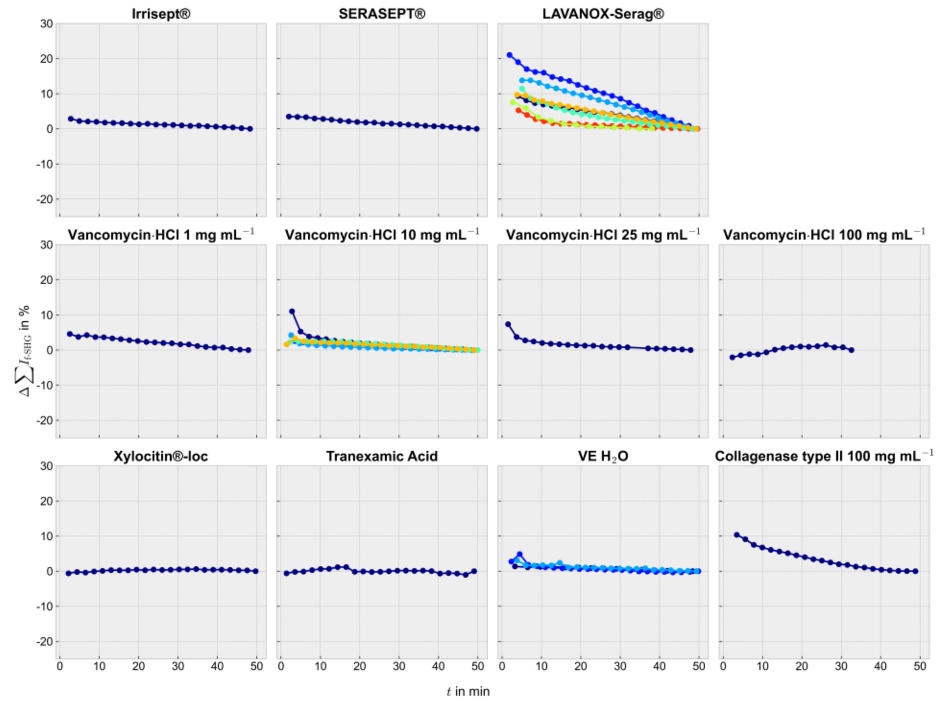

Figure S 7 Change of the cumulative f-SHG signal in the thin section volume of the dura mater section with respect to time after the addition of medications/media. For better visualization, the last measured point was used as a reference (0-line) and percentual changes were calculated with respect to this value. For more information on data evaluation, please refer to the main text.

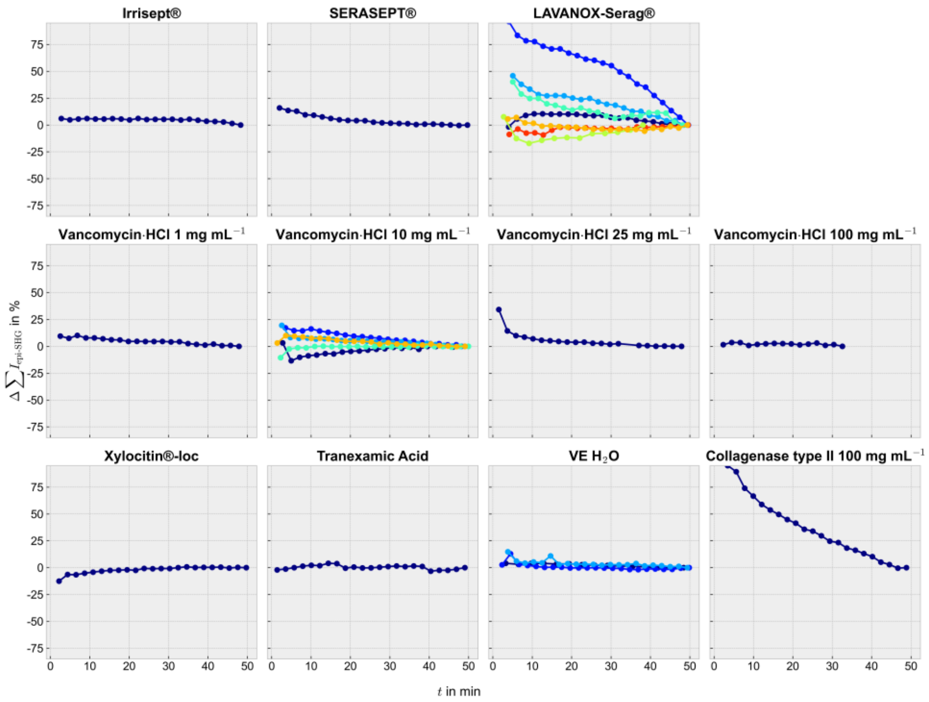

Figure S 8 Change of the cumulative epi-SHG signal in the thin section volume of the dura mater section section with respect to time after the addition of medications/media. For better visualization, the last measured point was used as a reference (0-line) and percentual changes were calculated with respect to this value. For more information on data evaluation, please refer to the main text.

1.2 2D-correlation maps between different features

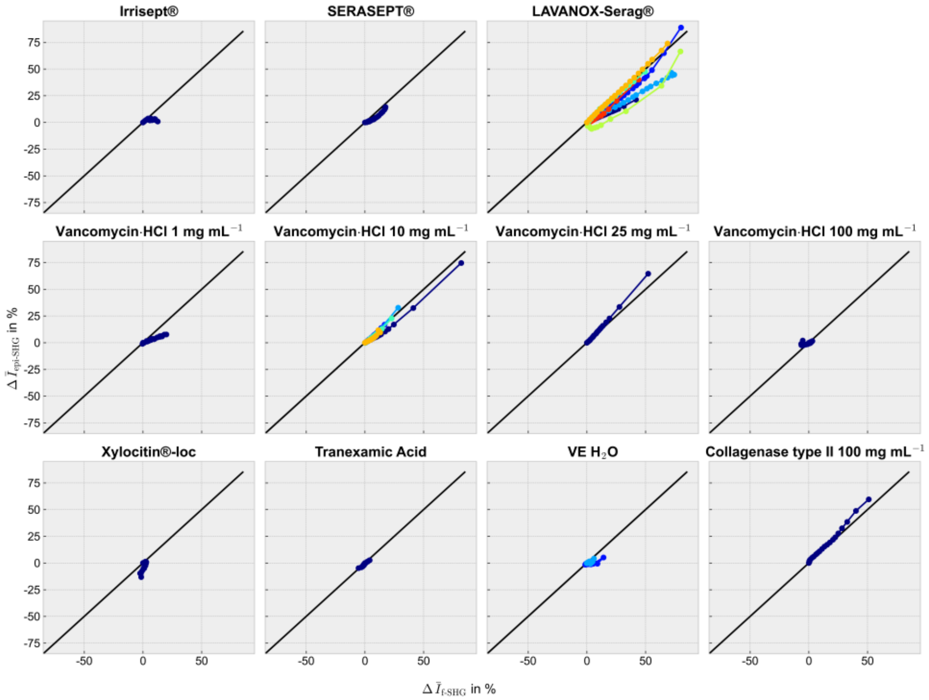

Figure S 9 Correlations between the average f- and epi-emitted SHG signal in the thin section volume during treatment with different medications/media. For better visualization, the last measured point was set into the origin of the plots and percentual changes were calculated with respect to this value. For more information on data evaluation, please refer to the main text.

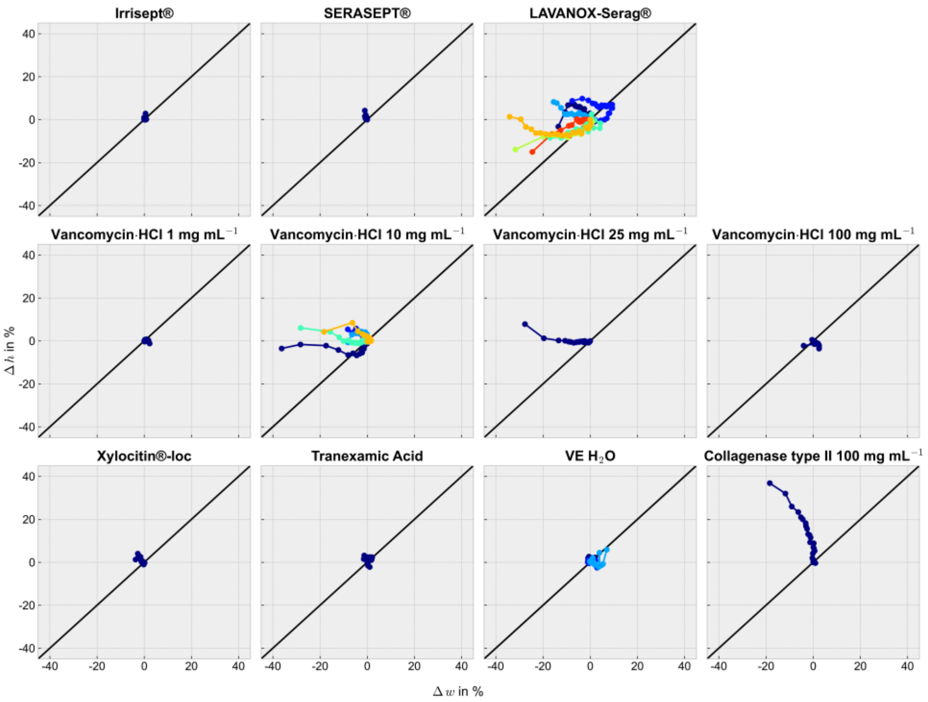

Figure S 10 Correlations between the thin section width and height during treatment with different medications/media. For better visualization, the last measured point was set into the origin of the plots and percentual changes were calculated with respect to this value. For more information on data evaluation, please refer to the main text.

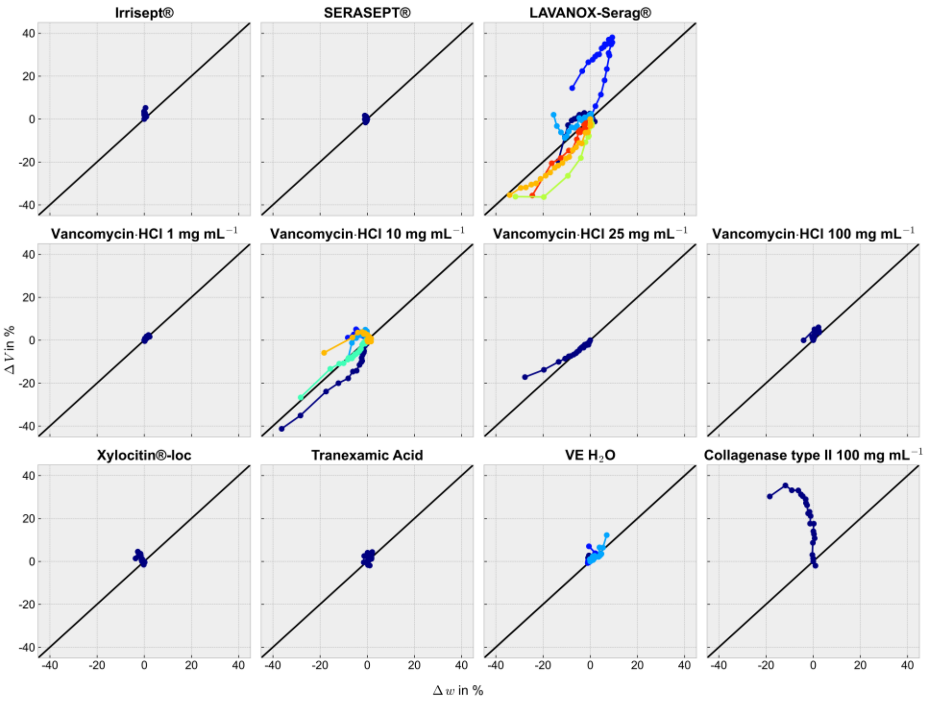

Figure S 11 Correlations between the thin section width and volume during treatment with different medications/media. For better visualization, the last measured point was set into the origin of the plots and percentual changes were calculated with respect to this value. For more information on data evaluation, please refer to the main text.

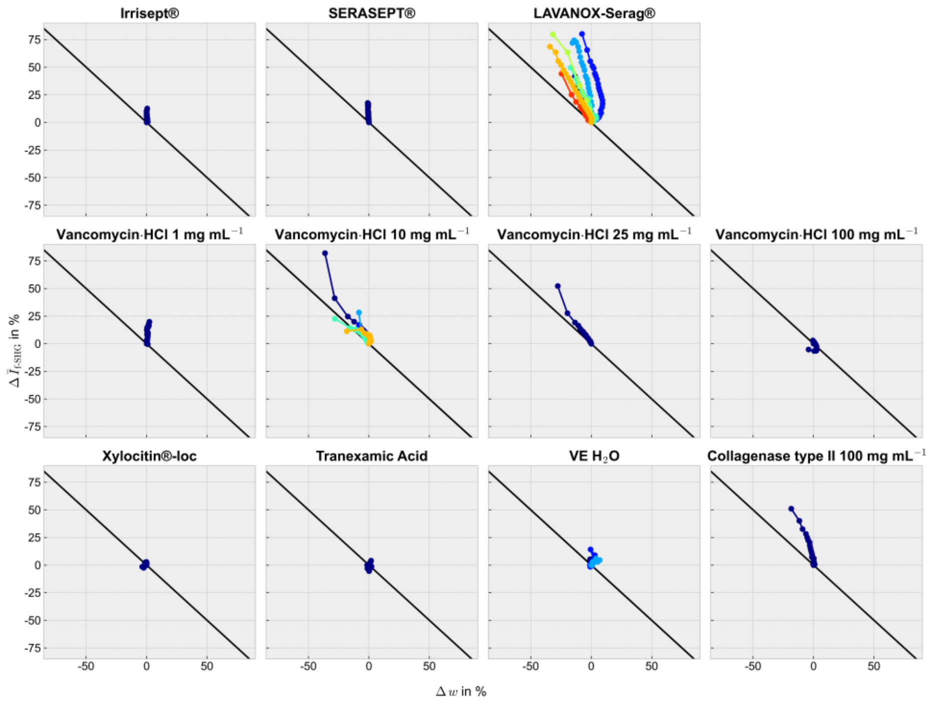

Figure S 12 Correlations between the thin section width and the average f-SHG signal in the thin section volume during treatment with different medications/media. For better visualization, the last measured point was set into the origin of the plots and percentual changes were calculated with respect to this value. For more information on data evaluation, please refer to the main text.

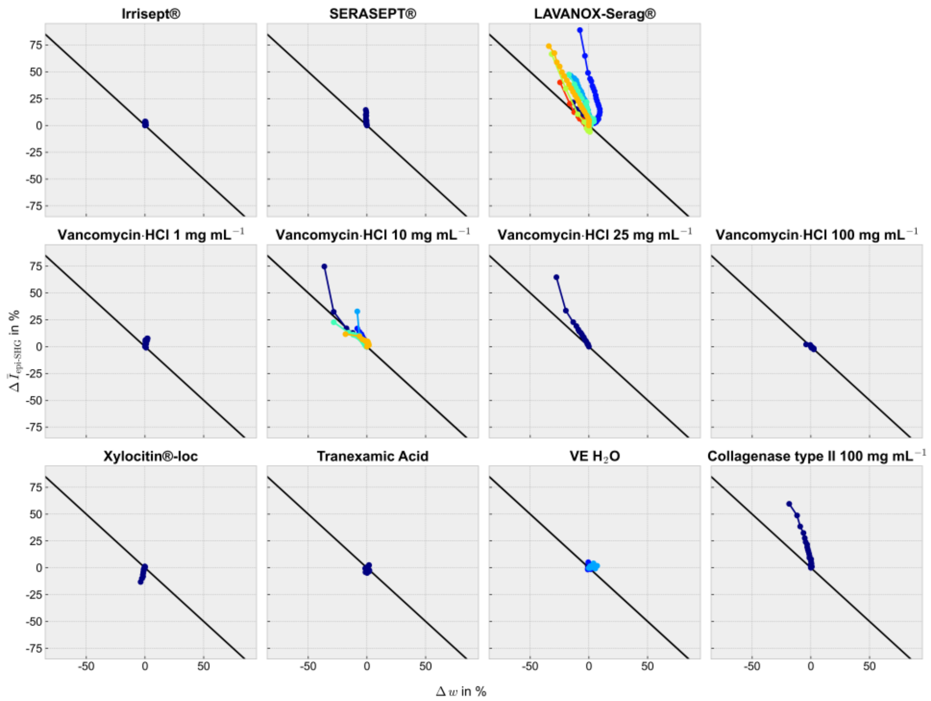

Figure S 13 Correlations between the thin section width and the average epi-SHG signal in the thin section volume during treatment with different medications/media. For better visualization, the last measured point was set into the origin of the plots and percentual changes were calculated with respect to this value. For more information on data evaluation, please refer to the main text.

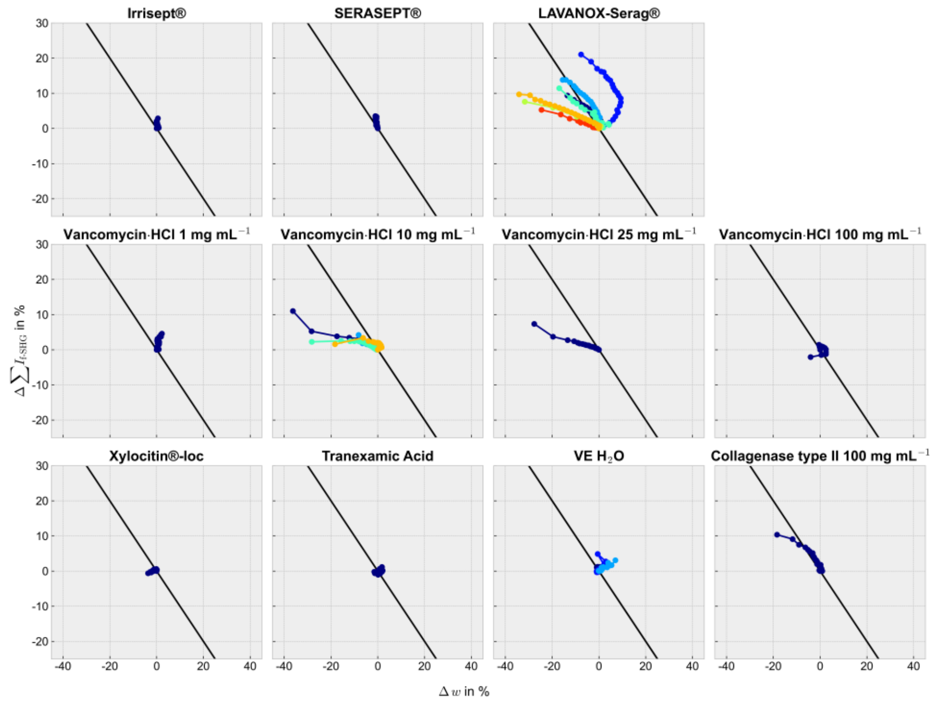

Figure S 14 Correlations between the thin section width and the cumulative f-SHG signal in the thin section volume during treatment with different medications/media. For better visualization, the last measured point was set into the origin of the plots and percentual changes were calculated with respect to this value. For more information on data evaluation, please refer to the main text.

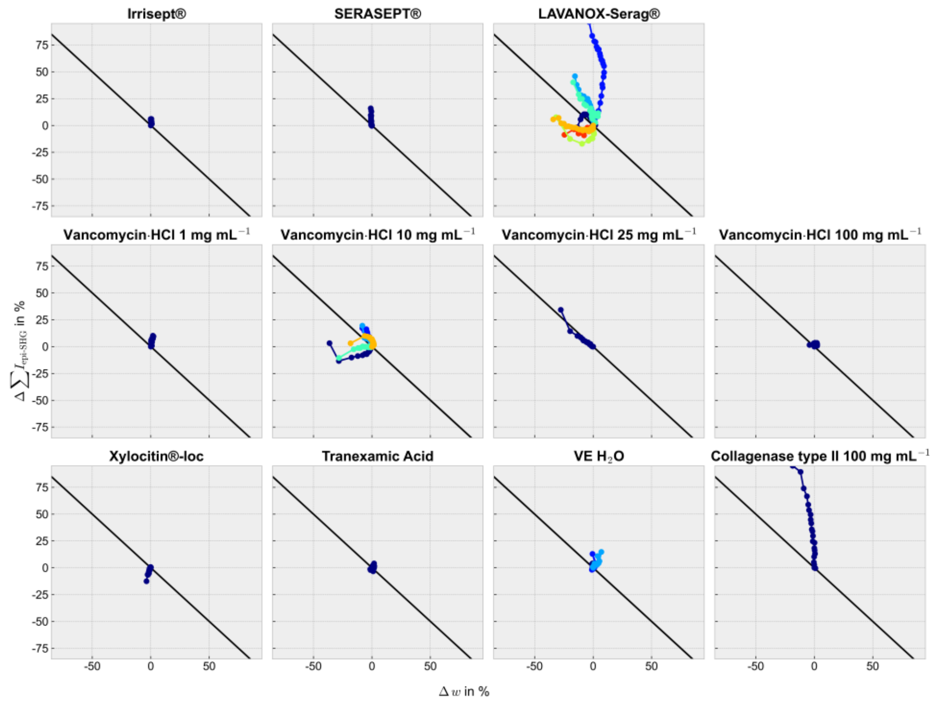

Figure S 15 Correlations between the thin section width and the cumulative epi-SHG signal in the thin section volume during treatment with different medications/media. For better visualization, the last measured point was set into the origin of the plots and percentual changes were calculated with respect to this value. For more information on data evaluation, please refer to the main text.

1.3 pH of the tested solutions

Rough pH values of the analyzed drugs were initially estimated using colorimetric pH indicator sticks (Roth) and subsequently more precisely obtained using a pH-sensing electrode (FiveEasyPlus pH/mV, Mettler Toledo, USA). The pH was recorded after the pH value was stabilized for 5 min. Table S1 lists the values obtained with the pH-sensing electrode. The measured values are in agreement with available medical data sheets. References from other scientifically independent studies concerning formulations with the same trade name or ingredient composition are given if applicable.

Table S 1: pH values of the tested drugs as determined by the pH-sensing electrode (pH(exp.)). Reference values are provided if available in the literature.

drug used	pH (exp.)	reference
VE H_2O	5.54	
LAVANOX-Serag	8.34	8.6[1]
Irrisept	5.49	5.0-7.0[2]
SERASEPT	5.77	5.5[3]
Xylocitin-loc 1%	5.79	6.09[4]
tranexamic acid	6.84	7.57[5]
vancomycin·HCl 1 mg mL^{-1}	4.21	3.72 (41.66 mg mL^{-1})[6]<br>3.57 (62.5 mg mL^{-1})[7]<br>3.54 (83.3 mg mL^{-1})[7]
vancomycin·HCl 10 mg mL^{-1}	3.44	
vancomycin·HCl 25 mg mL^{-1}	3.31	
vancomycin·HCl 100 mg mL^{-1}	3.21	

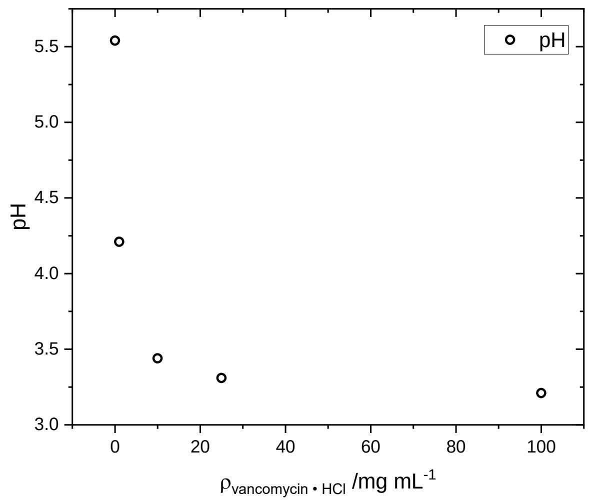

Figure S 16 Change of the pH of aqueous vancomycin hydrochloride solutions with concentration of vancomycin hydrochloride. As a solvent, VE H_2O was used.

2 Computational analysis of dimensional properties based on the forward-emitted SHG signal

2.1 Determination of the brightest plane

The determination of the plane of largest brightness was performed in *ImageJ* and is only based on the f-SHG (Figure S17A) channel as it shows larger and more well-defined signals than the channel recorded in the epi-direction. The aforementioned properties largely facilitate the distinction between the dura regions and the background. To obtain a result undisturbed by any signal absence in natural holes or tears (e. g. vessels) a hole filling and smoothing of the volume was executed first. For this purpose, a large 3D mean filter (*x*-radius =10.0 px, *y*-radius =10.0 px, *z*-radius =5.0 px) was applied which served both as the origin for the dura-background separation and for determining the plane of maximum intensity.

The segmentation of dura and background was performed by thresholding (Auto threshold, method triangle) of a sum projected image of the filtered stack. The thresholded image served as a mask later.

For the identification of the *z*-position of the pixel with the largest brightness at every *x*-*y*-combination, the following procedure was used.

1. The maximum intensity projection of the filtered stack was created. This projection shows the largest count with respect to the *z*-direction for every pixel position in the *xy*-plane
2. Division of the filtered stack by the maximum intensity image leads to a value of 1 at the pixel of maximum intensity and <1 for every other pixel in 32-bit mode.
3. For binarization in the categories "pixel of maximum intensity" and "other pixels", 1 was subtracted and absolute values were formed. Division of this stack by itself, multiplication with 255, and conversion to 8-bit led to an image of only values 0 or 255 whereas after inversion only the pixels of maximum intensity obtained the values 255.

The read-out of the *z*-position was done after the application of the mask to the binarized stack, setting all pixels in non-dura regions to 0. The *z*-position of the pixel of maximum intensity in the dura region was read out after reslicing (Figure S17B, red).

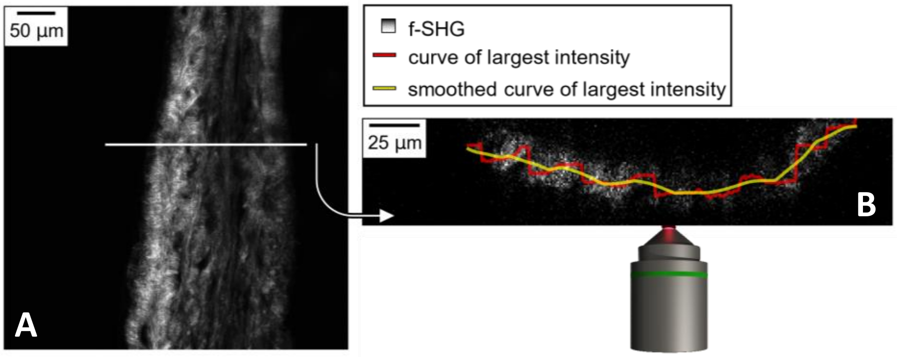

Figure S 17 Sum projection image of the f-SHG stack at one time point for a LAVANOX-Serag (10 mg / mL) treated dura thin section (A) and the orthographic view (B) at the indicated position in A. The red line in B corresponds to the determined position of the pixel of the largest brightness with respect to the z-direction after the application of the 3D mean filter. The yellow curve denotes the pixels of largest brightness after an additional smoothing operation. The smoothed curve runs parallel to the outer edges of the dura and thus can be used for determining of and correcting for dura bending.

The main dura piece was separated from loose pieces by keeping the largest region in 3D. If necessary additional manual adjustments by visual inspection were conducted.

A further smoothing operation was considered to be reasonable assuming a slowly changing position of the plane of greatest intensity with respect to the *z*-axis (Figure S17B, yellow). This time, a mean filter (quadratic kernel with 23 px width in x- and y-direction) was used in *Python*, thereby ignoring non-dura regions and ensuring no unwanted broadening of the dura signal region by averaging with the background. The remaining holes were filled by linear interpolation if necessary.

2.2 Determination of the average dura width

The plane denoting the pixel of largest brightness with respect to the z-axis served as the fundamental dataset for calculating the thin section width as it runs parallel to the outer borders of the thin section. It was determined as described in subsection 2.1 and represents the overall positioning of the thin section in space. Counting pixels in the x-direction for width determination is thereby an inadequate approach as bending and tilting in the xy- and xz-plane may falsify the result.

Error estimation for tilting in the *xy*-plane in dependence on the tilting angle α

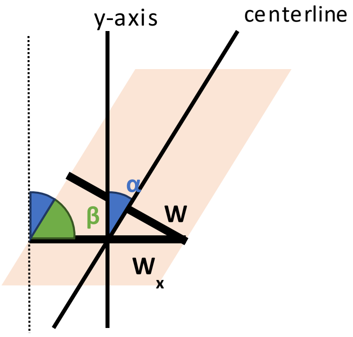

$$\frac{1}{\sin(90° - \alpha)} = \frac{w_x}{w}$$

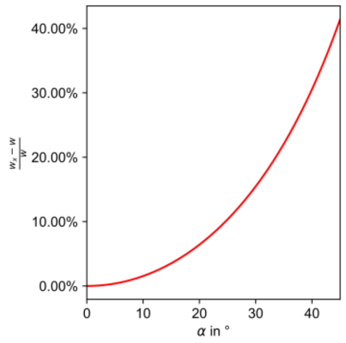

Error estimation for tilting in the *xz*-plane

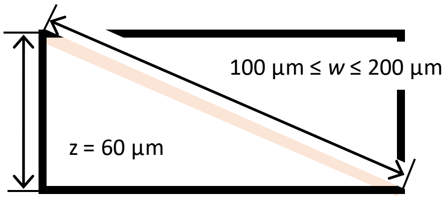

$$\frac{w}{w_x} - 1 = \frac{w}{\sqrt{w^2 - z^2}} - 1$$

$$3\% \leq \frac{w}{w_x} - 1 \leq 25\ \%, \qquad \text{für } 100\ \mu\text{m} \leq \text{w} \leq 250\ \mu\text{m}$$

Figure S 18 Error estimation for width calculation of a tilted dura section. **Top:** Estimation of the deviation of the width in the x-direction (w_x) and the true width (w) in dependence on the tilting angle (α) for the thin section (apricot) tilting in the xy-plane. Schematic drawing (left) and corresponding error plot (right) revealing errors >10 % for α > 25°. **Bottom**: Estimation of the maximum percentual error for the deviation of the width in the x-direction (w_x) and the true width (w) for a thin section that does not leave the imaging volume (black box). For error estimation, a thin section width between 100 µm and 250 µm and an imaging volume height of 60 µm were used.

The width of the dura was thus calculated as the length of the intersection line between the plane of largest brightness with respect to z and another plane being perpendicular to the course of the center line at a given point p. The workflow is shown schematically in Figure S19 and corrects for tilted and bent segments. This is particularly important since the position of the thin section may vary during and as a cause of the drug treatment. Consequently, this will otherwise lead to intricate results when comparing the width development as a function of time.

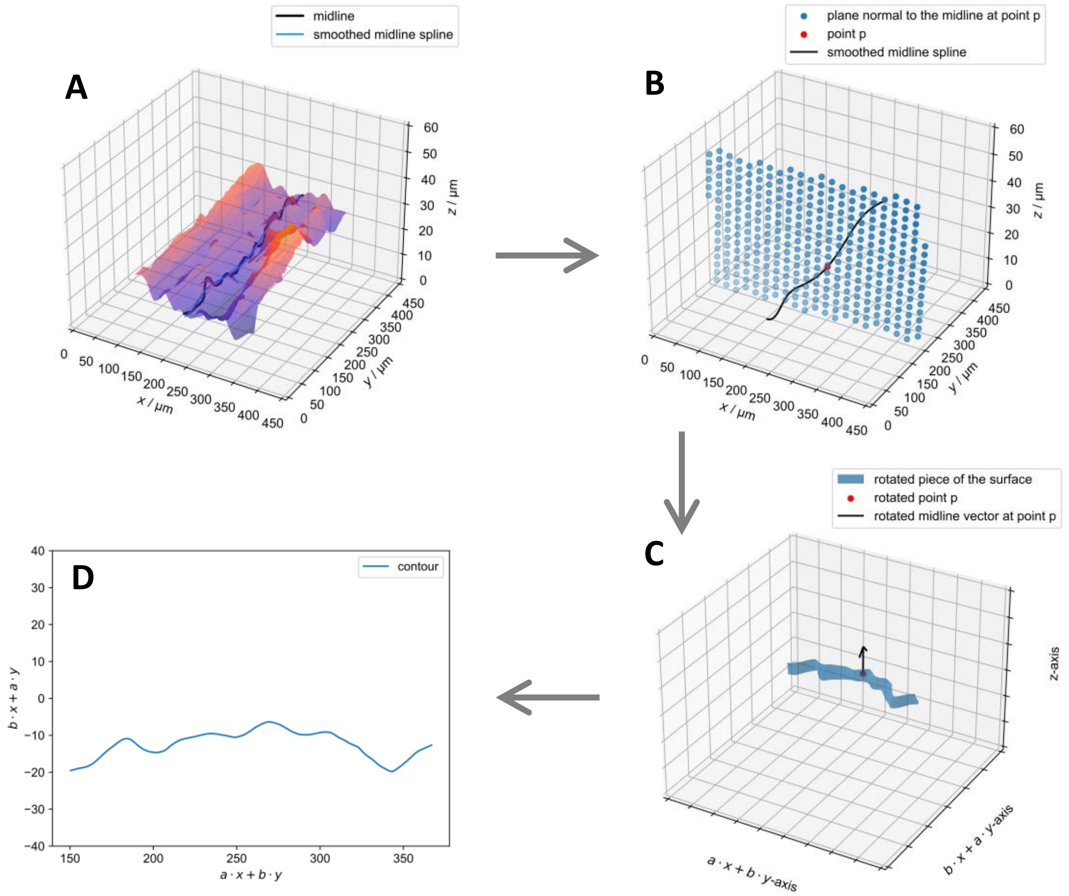

Figure S 19 Visualization of the workflow for width analysis taking a LAVANOX-Serag-treated thin section as an example. After calculating a smoothed centerline with respect to the plane of greatest brightness describing the orientation of the thin section in space (A), the slope of the centerline at each point *p* was used as a normal to determine a plane orthogonal to the thin section at point *p* (B). The sectioning line of this plane and the plane of largest brightness equals the width of an untilted thin section at that point. By rotating the surface so that the normal vector of the defined plane coincides with the *z*-axis (C), the true width can be evaluated using *Python*'s contour function and basic vector math (D).

The core element of this analysis is the midline spine that was derived from the pixel positions halving the curve $w_{x,}$ which is yielded from the intersection of the plane of largest brightness and a plane with the normal vector (0,1,0) located at position *y*. Subsequent smoothing by a rolling mean and by B-spline (degree 3) fitting resulted in a curve not following anymore strictly the calculated points but describing the rough course very well and coincidently removing leverages by spikes. In addition, the fitting procedure offers access to the 1st-order derivative at each point *p*, which is necessary for defining the normal vector of the intersection plane (blue in Figure S19B). Rotation of the dura segment in space so that the calculated normal vector runs parallel to the *z*-axis (Figure S19C) eventually allows a calculation of the dura width as the length of the intersection curve between the dura and a plane parallel to the *xy*-plane at $z = p_z$ (Figure 19D). Anisotropic voxel dimensions in the original coordinate systems were considered accordingly.

2.3 Volume reconstruction

The main difficulty in volume reconstruction is the smooth transition of the actual signal to the remaining signal of deeper focal planes/noise at the outer positions in the z-direction. Very good segmentation results were obtained by a 2-class classification on resliced f-SHG images by trainable WEKA segmentation[8]. The training data set included several *xz*-slices of a LAVANOX-Serag-treated sample (CS031D) from the first, twelfths, and last (23rd) recorded volume stack. The SHG intensities per time point were normalized (CLIJx 0.32.1.1)[9] and projected on a 16-bit scale before the segmentation. The training image, ground truth clusters, and the obtained classifier are provided as a part of the supplementary material. The segmented results were obtained as probability maps, allowing a user-specified threshold selection for distinguishing between the two categories of "dura" and "background" by visual inspection. The choice of common settings for thresholding and post-processing was based on two f-SHG images: one with very low signal strength (CS050A, time point 23, Figure S20 top right) and another one with an excellent signal-to-noise ratio (CS031D, time point 2, Figure S20 top left).

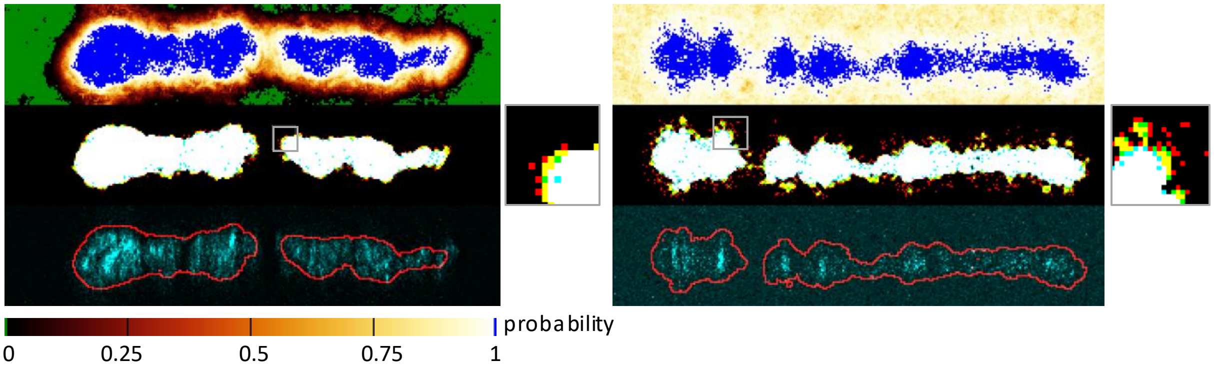

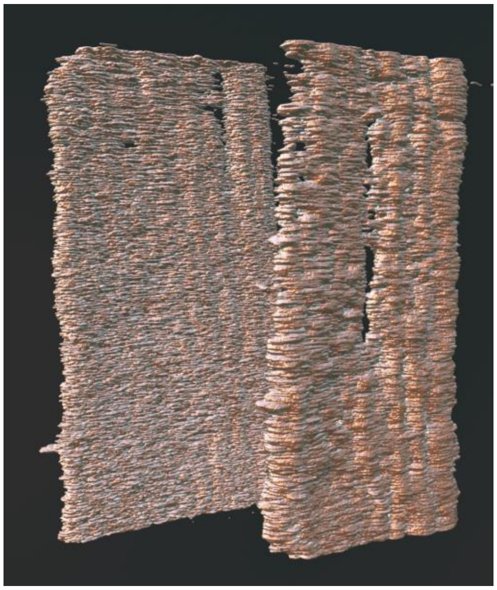

Figure S 20 ↑ Post-processing routine after WEKA segmentation for volume reconstruction. The procedure is shown for two samples with different f-SHG signal strengths (left: high SN-ratio, right: low SN-ratio) with the **top row** displaying the probability maps as the result of WEKA segmentation colored by likelihood value. The **middle row** compares different denoising operations to deal with salt and pepper noise in the dura and speckles at the borders (RGB image consisting of red channel: thresholded probability map, value = 0.99, green channel: thresholded probability map, value = 0.99 followed by *ImageJ's* despeckle algorithm, and blue channel: thresholded probability map, value = 0.99 followed by outlier removal (radius: 5 px) and despeckling). While all methods performed similarly for the high-signal dura, the latter method showed large improvements for the low-signal dura (cf. enlarged region). **Bottom row**: The resulting volume slices (red outline) are well in agreement with the original f-SHG data (cyan) for both test samples. ← 3D-reconstructed volumes for both samples (high-intensity sample at the front).

2.4 Determination of the average dura thickness

The dura thickness was calculated on every *xz*-slice of the segmented volume (cf. section 2.3 for segmentation and post-processing) as the pixel count in *z*-direction after straightening with *ImageJ*'s plugin "Straighten", using the smoothed curve of largest brightness (cf. section 2.1) as input for the segmented path. The described procedure corrects for bending and tilting in the xz-plane and ensures calculation of the height along the normal with the smoothed midline spline at every point *p*. The tilt in the *xy*-plane does not influence the height, while the tilt in the *yz*-plane was disregarded. The latter assumption was considered justified as the thin section length in the field of view (min. 450 µm) is much longer in comparison to the expected height (30 µm) which will cause only small errors for tilted states. In addition, as the thin section was fixed along its long axis, tilting in general becomes more unlikely in this direction. Artifacts resulting from straightening at the outer edges were neglected by ignoring 5 % of the total straightened slice width on the left and right sides, respectively. Only extracted widths with a value >1 µm contributed to the average width, intentionally ignoring background areas and holes. An example evaluation for a LAVANOX-Serag-treated dura thin section (CS031D) is shown in Figure S21).

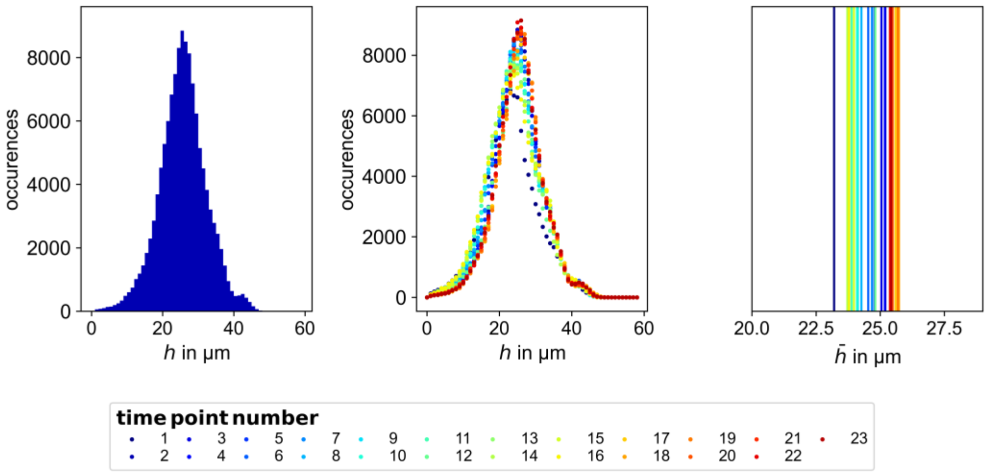

Figure S 21 Height development and evaluation for a LAVANOX-Serag-treated dura thin section over time. The extracted heights for one stack (>100000 data per time point) show a symmetric distribution (left), which keeps stable in its shape with time (middle). The mean values (right) of each time point's height distribution are similar and show only a slight shift over time that is not comparable with the significant changes observed in height.

3 References

(1) Severing, A.-L.; Rembe, J.-D.; Koester, V.; Stuermer, E. K. Safety and efficacy profiles of different commercial sodium hypochlorite/hypochlorous acid solutions (NaClO/HClO): antimicrobial efficacy, cytotoxic impact and physicochemical parameters in vitro. *J. Antimicrob. Chemother.* **2019**, *74*, 365–372.
(2) Razdan, S.; Siegal, A. R.; Celtik, K. E.; Carrion, R.; Valenzuela, R. J. Three piece penile prosthesis salvage with chlorhexidine gluconate and length preservation: our technique and outcomes. *Am. J. Clin. Exp. Urol.* **2023**, *11*, 155–159.
(3) Brill, F. H. H.; Hambach, J.; Utpatel, C.; Mogrovejo, D. C.; Gabriel, H.; Klock, J.-H.; Steinmann, J.; Arndt, A. Biofilm reduction potential of 0.02% polyhexanide irrigation solution in several types of urethral catheters. *BMC Urol.* **2021**, *21*, 58.
(4) Frank, S. G.; Lalonde, D. H. How acidic is the lidocaine we are injecting, and how much bicarbonate should we add? *Can. J. Plast. Surg.* **2012**, *20*, 71–73.
(5) Manosroi, A.; Podjanasoonthon, K.; Manosroi, J. Development of novel topical tranexamic acid liposome formulations. *Int. J. Pharm.* **2002**, *235*, 61–70.
(6) Godet, M.; Simar, J.; Closset, M.; Hecq, J.-D.; Braibant, M.; Soumoy, L.; Gillet, P.; Jamart, J.; Bihin, B.; Galanti, L. Stability of Concentrated Solution of Vancomycin Hydrochloride in Syringes for Intensive Care Units. *Pharm. Technol. Hosp. Pharm.* **2018**, *3*, 23–30.
(7) d'Huart, É.; Vigneron, J.; Charmillon, A.; Clarot, I.; Demoré, B. Physicochemical Stability of Vancomycin at High Concentrations in Polypropylene Syringes. *Can. J. Hosp. Pharm.* **2019**, *72*, 360–368.
(8) Arganda-Carreras, I.; Kaynig, V.; Rueden, C.; Eliceiri, K. W.; Schindelin, J.; Cardona, A.; Sebastian Seung, H. Trainable Weka Segmentation: a machine learning tool for microscopy pixel classification. *Bioinformatics* **2017**, *33*, 2424–2426.
(9) Haase, R.; Royer, L. A.; Steinbach, P.; Schmidt, D.; Dibrov, A.; Schmidt, U.; Weigert, M.; Maghelli, N.; Tomancak, P.; Jug, F.; Myers, E. W. CLIJ: GPU-accelerated image processing for everyone. *Nat. Methods* **2020**, *17*, 5–6.
