# Supplementary figures and images for: 4D-Spatiotemporal SHG Imaging for the Analysis of Drug-Induced Changes in the Dura Mater
Source: Anal Chem. 2025 Feb 14;97(7):3892–900. doi: 10.1021/acs.analchem.4c04887 (PMC11866281; doi:10.1021/acs.analchem.4c04887)

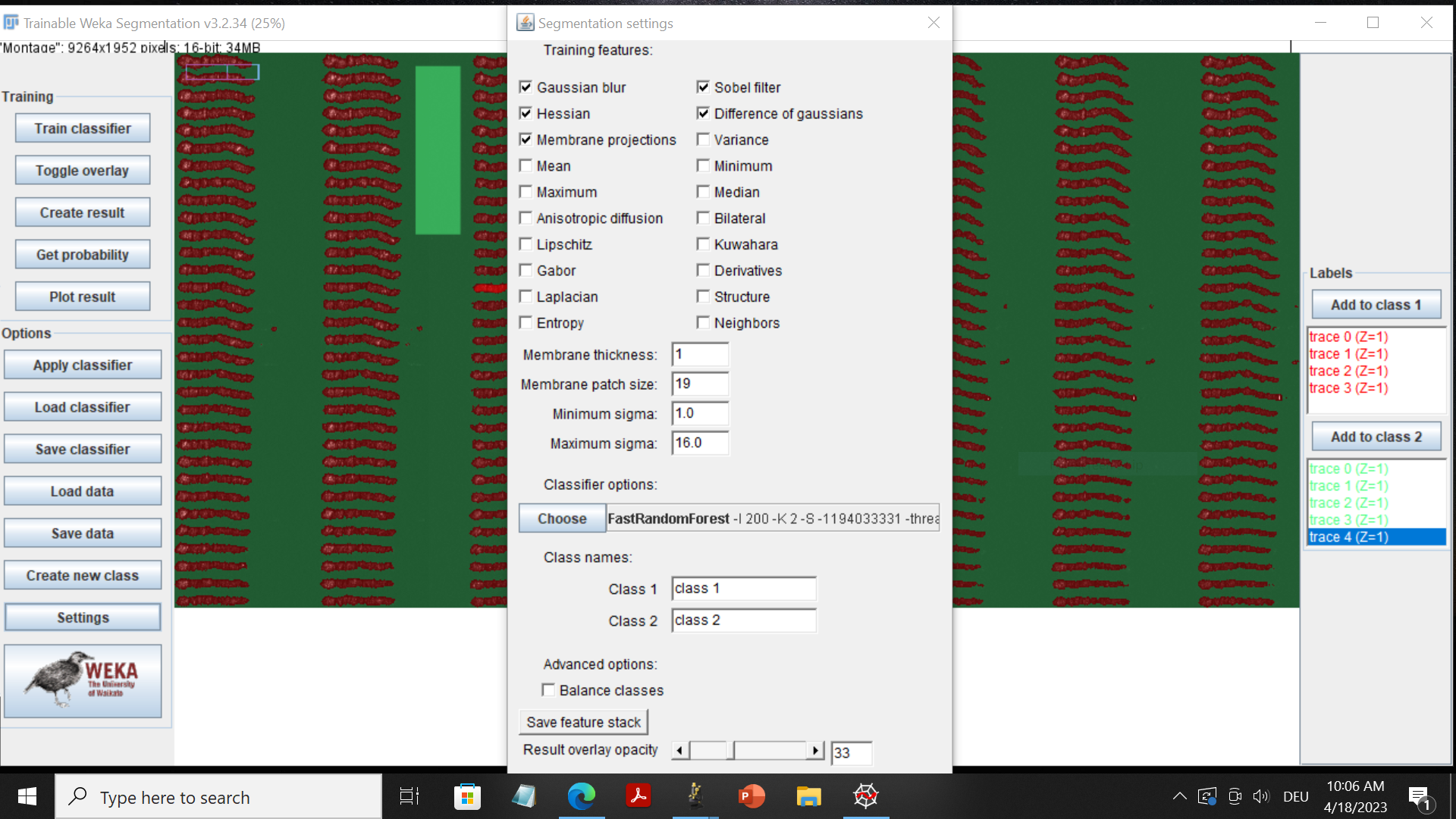

Supplement: Supplementary file 2 — ac4c04887_si_002.zip [file ac4c04887_si_002.zip › evaluation_scripts_and_architecture/volume/2_training/training_features_16bit.PNG]
